# The Dictionary of Virology, 4th Edition

**DOI:** 10.3201/eid1608.100778

**Published:** 2010-08

**Authors:** Aaron Brault

**Affiliations:** Centers for Disease Control and Prevention, Fort Collins, Colorado, USA

**Keywords:** Viruses, virology, dictionary, book review

Rapidly expanding technologies in the field of virology, identification of novel viral agents, and the 2005 report (8th edition) of the International Congress of Taxonomy of Viruses (ICTV) addressing reclassification of several viruses generated the 20% new material in Mahy’s 4th edition of The Dictionary of Virology. The previous edition of this book was published in 2001; the 2009 edition includes recent advancements, such as newly described viruses (e.g., severe acute respiratory syndrome (SARS) human coronavirus, human metapneumoviruses, bocaviruses, and Rabensburg virus), reclassification schemes of viruses (for instance, the unassigned *Anellovirus* genus), and descriptions of new technologies (e.g., microarray analyses and microRNAs) that have profoundly affected the field of virology. This comprehensive desk reference provides concise definitions of virologic terms; enables quick fact checking; and provides useful, often difficult to find, information—such as the origin of virus names, determination of ICTV-approved virus abbreviations, and locations and sources of viral isolations. An appendix of current ICTV-recognized virus families, subfamilies, genera, and type species is especially useful.

However, this reference is limited to viruses infecting vertebrate hosts; thus, it excludes viruses of plants, bacteria, fungi, invertebrates (except for arboviruses that have dual replication cycles within invertebrates and vertebrate hosts) or viruses (the newly described virophages of mimiviruses). The increasing quantity of information about viruses of vertebrates ranging from fish to primates presented the author with considerable space difficulties. He compensated for this situation, however, by citing literature sources at the end of entries for readers seeking more information. Additionally, considerable cross-referencing enhances the utility of the book. On the basis of inclusion of new information in the field and my personal experience with previous editions of The Dictionary of Virology, I highly recommend this volume to students, virologists, microbiologists, and public health professionals interested in viruses of vertebrate hosts.

